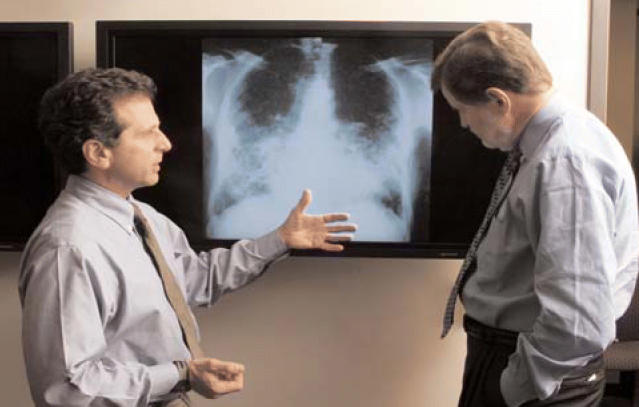# Disease-First: A New Paradigm for Environmental Health Science Research

**DOI:** 10.1289/ehp.114-1513300

**Published:** 2006-07

**Authors:** Samuel H. Wilson, David A. Schwartz

**Affiliations:** Deputy Director, NIEHS and NTP E-mail: wilson5@niehs.nih.gov; Director, NIEHS and NTP E-mail: david.schwartz@niehs.nih.gov

In recent years, many observers have advocated the adoption of a new paradigm in the environmental health sciences—a shift toward a sharper focus on understanding human disease and improving human health by integrating knowledge from environmental health research with that from the broad spectrum of medical research. We term this the “disease-first” approach. The first step in this approach is to prioritize specific common diseases according to the public health burden they pose. Next researchers will gather information on molecular changes that accompany the pathogenesis of each condition, including cellular and tissue changes that occur over time. We will then work to link these biological responses to environmental exposures including toxicants, metals, toxins, and lifestyle and dietary factors that eventually lead to disease. The fundamental goal of the NIEHS is to learn how this knowledge can be used to reduce morbidity and extend longevity. We believe the disease-first approach will allow our field to greatly reduce the burden of human disease.

The common diseases that account for the bulk of the public health burden of disease are chronic, disabling, and widely prevalent. Diseases such as asthma, obstructive lung diseases, and diabetes mellitus are leading causes of death in the United States and are increasing in incidence. These diseases are multifactorial and it is recognized that environmental exposures are a key risk factor in all of them, but large gaps exist in our ability to characterize the complex associations among environmental factors, genetic factors, and health outcomes. Gene–gene, gene–environment, and gene–vector–environment interactions all play important roles in any given condition. Thanks to advancing technologies in areas such as genomics, proteomics, and metabolomics, as well as in computational biology and bioinformatics, we now have the tools to redefine exposure–response relationships, develop quantitative models to support risk assessment, and reach more comprehensive understanding of the diverse and complex responses leading to pathogenesis. One of the key elements of the disease-first approach is to more fully leverage knowledge from these burgeoning technologies to vastly improve clinical outcomes.

The first priority of the disease-first approach is to more systematically track population health status in the United States, both temporally and spatially. Improved surveillance of health status can point to differences in exposures, which can be used as starting points for causality research. Although a comprehensive health status monitoring system does not yet exist, recent developments at the federal level suggest that it could soon be a reality [see Kyle et al., p. 980 this issue].

Such monitoring will allow for human body burden measurements of hazardous substances and xenobiotic metabolites. The disease-first approach will integrate this information with ongoing measurements from traditional environmental exposure assessments. These might include measurement of atmospheric pollutants and research on the fate and transport of hazardous agents in the ecosystem. The NIEHS will develop a number of exposure-based bioassays that will classify exposures (e.g., metals, chemicals, air pollution, diet) and link them to risk factors for developing clinical disease or to early steps in a disease process. Use of improved animal models of human disease will enable research on temporal aspects of dose–response relationships for harmful agents or lifestyle factors, facilitating the discovery of molecular signatures of thresholds separating the normal stress response from pathology. Such knowledge of thresholds will allow us to predict responses to stressors and to understand how individual susceptibility affects those responses.

We believe the disease-first approach will allow environmental health science to greatly reduce the burden of human disease.

Both population-based research and clinical research on individuals will be needed to ascertain correlations between “real world” exposures over ranges of dose, time, and disease, while accounting for genetic variations among subpopulations. By linking traditional exposure information with exposure responses, we can gain knowledge of diseases and early disease processes that will accelerate our understanding of both individual susceptibility and disease pathogenesis.

Many new tools will be required to accomplish these goals, some of which are now becoming available and others of which will soon be possible. We have seen the rapid maturation of novel, high-throughput analytical methodologies in the various “-omic” sciences, as well as advances in nanotechnology, imaging, and bioinformatics. Development in these areas will continue with support from both the public and private sectors. Improved understanding of disease at the molecular level and of pathophysiologic processes, along with research innovations such as RNA interference and subcellular imaging, will allow more precise analysis of mammalian pathophysiology. Geographic information systems and sensor-based technologies will enable greater precision in environmental and personal exposure monitoring, integrating previously unavailable data into research.

The NIEHS research portfolio will emphasize understanding of molecular mechanisms of pathogenesis and directly link environmental exposures with common diseases. We have established a new Office of Translational Biomedicine at the NIEHS to facilitate interdisciplinary and cross-disciplinary research endeavors, and we will seek to collaborate more broadly across the medical research community to maximize the potential gains from this approach.

This new paradigm for research in the environmental health sciences is our challenge and our vision for a healthier future.

## Figures and Tables

**Figure:**